# Efficacy of Virucidal Treatments against Poliovirus Type 1 Deposited on Stainless-Steel Carriers

**DOI:** 10.3390/microorganisms12101964

**Published:** 2024-09-27

**Authors:** Cory Chiossone, Tanya Kapes, Robert Good, Raymond W. Nims, S. Steve Zhou

**Affiliations:** 1 Microbac Laboratories, Inc., Sterling, VA 20164, USA; cory.chiossone@microbac.com (C.C.);; 2 Syner-G Biopharma, Boulder, CO 80503, USA; ray.nims@synergbiopharma.com

**Keywords:** Cavicide inactivation, combined UV-C/chemical inactivation, ethanol inactivation, sodium hypochlorite inactivation, poliovirus type 1 Chat strain, surface inactivation

## Abstract

Safe containment of infectious poliovirus (PV) within Poliovirus-Essential Facilities (PEFs) will require the implementation of reliable PV-inactivation approaches for decontaminating work surfaces. Such approaches should be demonstrated empirically to display adequate efficacy at the use temperature, and the contact times required should be characterized to ensure efficacy. Such efficacy is judged by the ability of the inactivation approach to completely inactivate any PV deposited, with the demonstrated total log_10_ reduction in PV titer being as high as empirically achievable. We screened several approaches for their efficacy in inactivating wild-type PV type 1 Chat strain experimentally deposited on stainless-steel carriers at room temperature. On the basis of the results, we selected two approaches (5000 ppm sodium hypochlorite in water and 95% *v*/*v* ethanol in water) for further characterization for repeatability of efficacy (log_10_ reduction in PV titer) and time kinetics of inactivation. We now report that both PV-inactivation approaches, which should be readily available to all PEF laboratories globally, fulfill the expectations expressed above, with 5000 ppm sodium hypochlorite reproducibly causing ≥5.38 log_10_ inactivation and 95% ethanol reproducibly causing ≥4.46 log_10_ inactivation of PV on stainless-steel surfaces within a 5 min contact time at room temperature.

## 1. Introduction

A global effort aimed at eradicating infectious poliovirus (including types 1, 2, and 3, as well as variant vaccine-derived strains) by 2026 is currently underway [1]. The eradication effort has been undertaken with the goal of globally preventing further cases of acute flaccid paralysis (also referred to as acute flaccid myelitis, and in the past, as infantile paralysis) that is caused in certain individuals infected with this virus. Poliovirus (PV) is a member of the *Picornaviridae* family and is a small-sized (~30-nm) non-enveloped RNA virus. An important aspect of the eradication effort consists of efforts intended to safely contain infectious PV within designated research and diagnostic laboratories worldwide and to limit the possibility of unintended releases of infectious PV to the environment and human population. The proposed PV-containment requirements are addressed in the World Health Organization’s Global Action Plan for Poliovirus Containment 4th Edition (GAPIV) [2]. Moving forward, infectious PV will be contained (stored, manipulated, etc.) only in Poliovirus-Essential Facilities (PEFs) [2]. The requirements of the GAPIV are necessary since “A loss of containment and breach in biosafety at a PEF can lead to an outbreak of poliovirus that could threaten global eradication.” [2].

An important aspect of the containment strategy involves identifying reliable methods for inactivating any infectious PV currently present in non-PEF sites, as well as inactivating PV in PEF sites prior to release to the environment. Such inactivation methods should be shown to provide complete inactivation at empirically determined concentrations and have contact times that provide “overkill conditions” designed to provide an adequate safety margin. The importance of this stems from the fact that viral stocks often contain infectious viral titers exceeding 10^6^ tissue culture infectious dose_50_/mL. Thus, the goal of inactivation approaches for PV inactivation in PEF and non-PEF sites will be to exceed the typical U.S. Environmental Protection Agency (EPA) disinfectant efficacy registration claims [3] requiring >3 log_10_ inactivation.

In this project, we screened several commonly used chemical and chemical/physical inactivation approaches for efficacy against PV type 1 (Chat strain) experimentally deposited on stainless-steel carriers, using the ASTM E1053-20 standard modified for stainless-steel surfaces [4]. Stainless steel was selected as the carrier type for these studies since this represents a common surface used for manipulating infectious viruses (e.g., within a biosafety cabinet). For those PV-inactivation approaches found to display the required efficacy as surface-disinfecting agents, we have characterized the time kinetics and repeatability of PV inactivation to establish contact time recommendations that should provide the required margin of safety for the described infectious PV-containment application.

## 2. Materials and Methods

### 2.1. Challenge Virus and Host Cell Line

Poliovirus type 1 strain Chat (ATCC VR-1562) was sourced from American Type Culture Collection (Manassas, VA, USA). The host cell line used for propagating and detecting the virus was African green monkey fetal kidney (MA-104), originally obtained from Charles River Laboratories, Inc. (Wilmington, MA, USA). The cell culture medium used for the MA-104 cell line was RPMI 1640 (Thermo Fisher Scientific, Waltham, MA, USA) + 10% fetal bovine serum (FBS, Cytiva Life Sciences, Marlborough, MA, USA). The virus stock was prepared as follows: PV was diluted in RPMI + 5% FBS and added to T-75 flasks of MA-104 cells. The flasks were incubated at 36 °C for 90 min to allow for viral adsorption after which they were refed with RPMI + 5% FBS. The flasks were incubated at 36 °C until ~ 90% of the cells exhibited a viral cytopathic effect (CPE). At that time, the flasks were frozen at −80 °C and then thawed at ambient temperature three times. The conditioned medium containing PV was collected from the flasks and clarified at 2000 rpm for 15 min, and the resulting supernatant was aliquoted and stored at −80 °C until use. The certified titers of the PV stocks were determined to be 8.12–8.50 log_10_ tissue culture infectious dose_50_ per mL (TCID_50_/mL) in MA-104 cells.

### 2.2. Microbicides and Neutralizers Used

The microbicidal chemicals evaluated in this study included sodium hypochlorite (Clorox Germicidal Bleach, Clorox, Oakland, CA, USA), ethanol (Fisher Scientific, Hampton, NH, USA), and Cavicide^®^ (active ingredients: the quaternary ammonium compound benzethonium chloride at 0.38% and isopropyl alcohol at 17.2%) (Henry Schein, Melville, NY, USA). Sterile deionized water was used to prepare dilutions to use concentrations (80% *v*/*v* or 95% *v*/*v*) for ethanol. The chemical neutralizer used to quench the virucidal activities of the active ingredients at the end of the contact times consisted of Minimum Essential Medium (MEM, (Thermo Fisher Scientific) + 10% newborn calf serum [NCS, (Thermo Fisher Scientific] + 0.5% Polysorbate 80 (Fisher Scientific, Waltham, MA, USA) + 0.5% Na_2_S_2_O_3_ (thiosulfate, Hach Company, Loveland, CO, USA). The methods used to determine the potential for detector cell cytotoxicity and inhibition of viral proliferation due to the chemical neutralizer used are described in the Supplementary Materials.

### 2.3. Surface (Carrier) Testing Methodology

Surface (carrier) viral inactivation studies were performed per the ASTM E1053-20 standard with a modification to use stainless-steel carriers [4]. For each run, a 2-inch × 2-inch square stainless-steel plate was inoculated with 0.4 mL of virus stock and allowed to dry. The virus-contaminated carriers then were treated either with 2.0 mL of microbicide via a direct soak or via a spray with approximately 2.0 mL of microbicide in the presence or absence of UV irradiation (see Table 1). UV-C (254 nm) irradiation was performed within a class II biosafety cabinet, with the distance between the UV-C lamp and the virus being ~24 inches (~61 cm, see Table 1). After treatment, the carriers were held for the stipulated contact times. Virus then was recovered from the carriers using 2.0 mL of the chemical neutralizer described above. The recovered surface samples were serially diluted in dilution medium (MEM + 2% NCS) and assayed using a Tissue Culture Infectious Dose 50% (TCID_50_) assay using MA-104 cells as detector cells. The inoculated host cells were incubated at 36 °C with 5% CO_2_ for 5–11 days and scored for viral cytopathic effects. The infectious virus titers were calculated using the Spearman–Kärber formula [5]. For the Plate Recovery Control, the same procedure was used as for the test runs, except that the virus-contaminated carrier was treated with dilution medium instead of test microbicide. A neutralizer effectiveness control and a cytotoxicity control were also performed by treating carriers upon which cell culture medium had been deposited in lieu of virus (see Supplementary Materials). All inactivation treatments and holding were performed at an ambient temperature (20 ± 2 °C).

The inactivation results from the time kinetics study for 95% ethanol were analyzed, where appropriate, by a two-tailed T-test to determine the statistical significance of differences in the log_10_ reduction obtained after the varying exposure times for ethanol treatment. To enable these comparisons, all log_10_ reduction values were considered complete. No statistics were performed for the sodium hypochlorite time kinetics study since each of the time points yielded complete inactivation.

## 3. Results

In the first phase of this study, several candidate approaches were evaluated for efficacy in inactivating PV deposited on stainless-steel carriers at room temperature. On the basis of the results obtained in the screening portion of the study, two inactivation approaches were selected for more detailed evaluations for contact time kinetics and repeatability.

### 3.1. Evaluation of Cytotoxic Effects of the Microbicides + Neutralizer

The results of the evaluation of the cytotoxic effects of the test microbicides and the neutralizer used to quench the virucidal activity of the test microbicides are displayed in Supplementary Materials Table S1. Any cytotoxic effects of the mixture of microbicide + chemical neutralizer on the host cells, if found to be present, have been factored into the final log_10_ reduction results presented below.

### 3.2. Initial Screening Evaluation of Inactivation Approaches for Poliovirus Type 1 Chat Strain

The PV-inactivation approaches selected for initial efficacy screening are displayed in Table 1. They involved disinfection agents/modalities commonly available in virology laboratories. The germicidal UV-C lamps contained within many biosafety cabinets are used to help maintain aseptic conditions within the cabinets. An approach for routine biosafety cabinet disinfection involving a 15 min UV-C exposure, followed by a 5 min soak with Cavicide, followed by a spray and wipe with 70% isopropyl alcohol was found (Table 2) to only be minimally more effective than a 5 min soak with Cavicide alone. The incomplete (<3 log_10_) inactivation of PV deposited on stainless-steel carriers afforded in either case was not deemed adequate for further study.

A dose–response study of sodium hypochlorite applied as a 5-min soak suggested that 1000 ppm was inadequate, leading to incomplete (2.00 log_10_) inactivation of PV (Table 2). On the other hand, the 5000 and 10,000 ppm sodium hypochlorite soaks applied for 5 min achieved complete inactivation (≥4.50 and ≥3.75 log_10_, respectively) (Table 2). The 5000 ppm hypochlorite condition was selected for further evaluation. Ethanol was also applied as a 5-min soak at 80% or 95% strength. The 80% strength ethanol caused incomplete (2.00 log_10_) inactivation of PV, which was not deemed adequate. The 95% ethanol caused complete inactivation and was selected for further evaluation. Evaluation of 95% ethanol solution aged for 30 days (Table 2) demonstrated incomplete (2.35 to 2.60 log_10_) PV inactivation for the 1 min contact time and complete (≥4.25 log_10_) PV inactivation for the 5-min contact time.

### 3.3. Time Kinetics of the Inactivation of Poliovirus Type 1 Chat Strain on Stainless-Steel Carriers by Sodium Hypochlorite and Ethanol at Room Temperature

**Figure 1:**
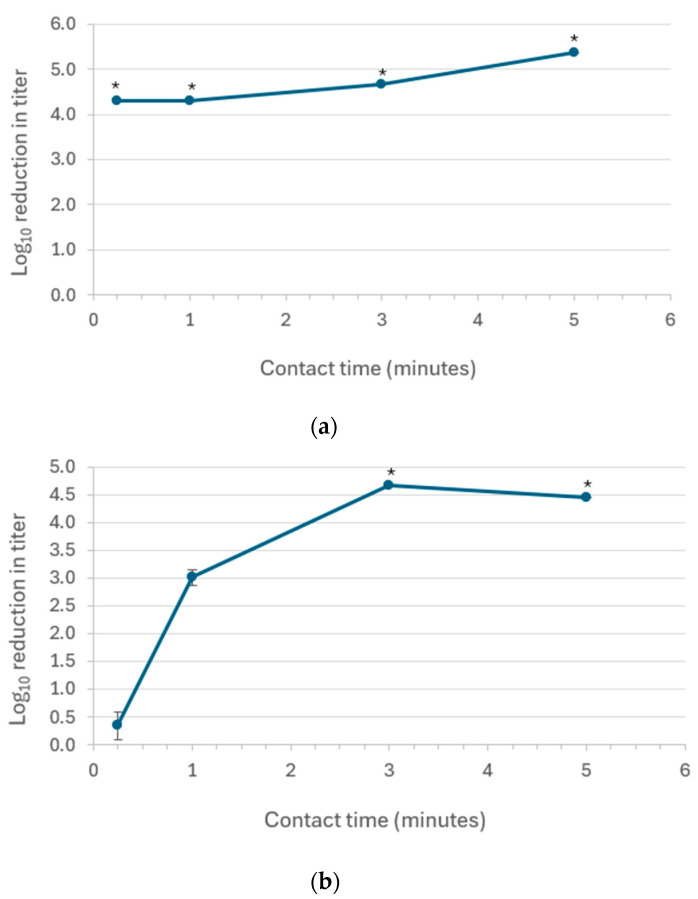
Time kinetics of the inactivation of poliovirus type 1 Chat strain on stainless-steel carriers at room temperature by (**a**) 5000 ppm sodium hypochlorite; and (**b**) 95% *v*/*v* ethanol. The values shown are the mean ± standard deviation for *n* = 3 independent replicate runs. * Inactivation was complete (no residual infectious virus was detected), and, therefore, the log_10_ reductions depicted are to be interpreted as “≥” the values shown.

From the screening portion of the study described above, 5000 ppm sodium hypochlorite and freshly prepared 95% ethanol were selected for further evaluation to assess the time kinetics of inactivation and repeatability for inactivating PV experimentally deposited on stainless-steel carriers. The experiments followed the same methodology used for the screening studies but used fixed microbicide concentrations while varying the contact times. The PV-inactivation time kinetics for 5000 ppm sodium hypochlorite and 95% ethanol are displayed in Figure 1a,b, respectively, with additional experimental detail provided in Table 3.

The results demonstrated that the inactivation of PV by 5000 ppm sodium hypochlorite was complete by 0.25 min (15 s) for each of the three independent replicates evaluated (Figure 1a, Table 3). The log_10_ reductions achieved at the various contact times differed only due to differences in the starting PV titers and the limits of detection of the titration assay (Table 3). The highest calculated log reduction (≥5.38) was obtained for the 5-min contact time (Table 3). In the case of 95% ethanol, a clear dependence on contact time was observed, with PV inactivation being incomplete for the 0.25- and 1-min contact times and complete and reproducible for the 3- and 5- min contact times (Figure 1b, Table 3). The log_10_ reductions achieved for the 3- and 5- min contact times differed only due to differences in the starting PV titers and the limits of detection of the titration assay (Table 3).

## 4. Discussion

One of the aspects of future containment strategies [2] for PV will be to ensure that any PV inadvertently deposited on surfaces during manipulation of the virus can be completely inactivated during decontamination of the surfaces. One of the most likely scenarios might be the inadvertent contamination of the stainless-steel work surface of a biosafety cabinet during the manipulation of cultures or virus stocks. A requirement for the approval of PEF sites in the future will be the identification within those facilities of empirically verified approaches for decontaminating PV-contaminated environmental surfaces. The exact wording from the GAPIV [2] is that decontamination SOPs must be “validated in-house and shown to be effective against poliovirus prior to their use. The validation process includes the use of positive and negative controls”. Though not stated explicitly in the GAPIV, the goal of inactivation approaches for PV inactivation in PEF sites should be to exceed the typical U.S. Environmental Protection Agency (EPA) [3] and Health Canada [6] disinfectant efficacy registration claims requiring ≥3 log_10_ inactivation. This is because, as mentioned previously, the titers of PV virus stocks being manipulated at PEF sites may exceed 10^6^ TCID_50_/mL.

One might ask the following question: in the absence of a decontamination strategy, how long will PV remain infectious once spilled onto a work surface? As with other small non-enveloped viruses, PV deposited on an environmental surface can remain infectious for an extended period of time, depending primarily on the initial virus titer deposited, the type and porosity of the surface, and the temperature and relative humidity (RH) [7,8,9,10,11]. This period may amount to several weeks to months for enteric viruses such as PV [11]. Persistence of viruses on surfaces is often reported as half-lives or as the time required to achieve a 1- or 2-log_10_ reduction in titer. As such, the duration of viral persistence is dependent upon the initial contaminating titer.

For instance, Abad et al. [7] evaluated the persistence of infectious PV type 1 LSc 2ab strain in a phosphate-buffered saline (PBS) matrix as well as in a 20% fecal suspension (FS) on porous and non-porous surfaces under conditions of differing RH and temperature. PV infectivity persisted longer (persistence for at least two months) at 4 °C than at 20 °C (persistence for <10 days at high or moderate RH), and on non-porous surfaces (polystyrene plastic, aluminum, china, glazed tile, and latex) than on porous surfaces (cotton cloth and paper). When deposited on non-porous surfaces, PV persistence was longer in the FS matrix than in PBS. The opposite was true for porous surfaces [7]. Tuladhar et al. [9] evaluated the persistence of PV type 1 Sabin strain on plastic and glass surfaces. In this experiment 8 × 10^5^ TCID_50_ PV was deposited on the carriers. Less than a 1 log_10_ reduction in titer was observed over the following 10 days at room temperature [9]. Tamrakar et al. [10] studied the persistence of infectious PV type 1 LSc 2ab strain on stainless-steel, cotton, and plastic laminate coupons at room temperature. As in the Abad et al. study mentioned above [7], Tamrakar et al. observed rapid initial PV decay on all fomite types, followed by more gradual decay over the remaining time up to three weeks [10]. Times required for a 99% (2-log_10_) attenuation in titer were 102 h (stainless steel), 143 h (cotton), and 81 h (plastic laminate) in the Tamrakar et al. study [10]. A review of pathogen persistence on inanimate surfaces by Wißmann et al. [11] may be consulted for results of additional studies on PV persistence on environmental surfaces. Taken together, these persistence data inform the need for effective inactivation approaches for PV that might be inadvertently spilled onto work surfaces.

Our initial screening of candidate surface PV-inactivation approaches indicated that the quaternary ammonium compound (QAC) formulation Cavicide displayed insufficient inactivating activity (2.00 log_10_) after a 5-min contact time (Table 2). This result is not unexpected as Tuladhar et al. [9] reported previously that a variety of QAC coatings on glass and plastic displayed less than a 1 log_10_ reduction in PV Sabin 1 vaccine strain at room temperature. In addition, Nims and Zhou [12] reported incomplete (less than 3 log_10_) surface inactivation of PV type 1 after a 2- or 10-min contact time with two proprietary QAC formulations at room temperature. The efficacy of the Cavicide soak in the present study was only marginally improved (i.e., to 2.82 log_10_) when preceded by a 15-min exposure of the surface to UV-C light. The 0.82-log_10_ additional inactivation afforded by the UV-C light exposure (calculated to result in a fluence of 139 mJ/cm^2^) was surprisingly low. Suspension inactivation data from Simonet and Gantzer [13] demonstrated linear inactivation curves through 60 mJ/cm^2^ with tailing off through the virus detection threshold through 125 mJ/cm^2^. We are not aware of the existence of previous reports of UV-C efficacy for the surface inactivation of PV.

Ethanol at 80% *v*/*v* did not represent a PV-inactivation approach with adequate efficacy in our screening study, as a 5-min contact time led to only a 2.00 log_10_ reduction in titer (Table 2). Tyler et al. [14] also found that 80% ethanol led to a less than 3 log_10_ inactivation of PV Sabin type 1an on glass coverslips at room temperature after 1 or 5 min. We found that 95% ethanol, either freshly prepared or aged for 30 days, caused complete inactivation of PV after a 5-min contact time (Table 2). The 1-min contact time caused incomplete inactivation. The 95% ethanol approach was selected for further evaluation. The complete inactivation of PV within 5 min found in our study differed from the results of Tyler et al. [14], who found that both 90% ethanol and absolute ethanol caused only incomplete (≤2.3 log_10_) inactivation of PV Sabin type 1an on a glass surface within a 1-, 5-, or 10-min contact time. The difference in results may have been due to methodological differences as the Tyler et al. work involved a rinsing step in neutralizer prior to the recovery of residual infectious virus from the glass coverslips

We did not explore peracetic acid-, aldehyde-, or hydrogen peroxide-based microbicides for surface PV-inactivation efficacy in the screening portion of the present study. These approaches have been evaluated in previous PV-inactivation studies [12,13,14,15,16,17,18]. Microbicides formulated with 0.25% glutaraldehyde or 0.075% peracetic acid have been found to be effective (3–6 log_10_) against PV type 1 strain LSc 2ab when tested in suspension with a 30-min contact time [16], and microbicides formulated with 0.80–0.85% peracetic acid tested in stainless-steel carrier studies using a 10-min contact time have also been found to be effective (4 log_10_) [15]. Two proprietary aldehyde-based products (Microbide-S and Microbide-G) were found to cause complete (≥4 log_10_) inactivation of PV-1 strain Chat on a glass surface at concentrations ≥15,000 ppm and contact times ≥ 1 min [17].

Two approaches for inactivating PV on stainless-steel surfaces were selected for time kinetics and repeatability evaluation in the second phase of our study. Sodium hypochlorite (the active ingredient contained in household bleach) at 5000 ppm was found to lead to complete inactivation (≥4.3 to ≥5.4 log_10_) of PV at the contact times evaluated (0.25, 1, 3, and 5 min) (Figure 1a and Table 3). This result is not unexpected as Orel et al. [18] found that sodium hypochlorite at 0.63% (6300 ppm) caused complete inactivation (≥7.2 log_10_) of PV type 1 (Mahoney strain) and (≥5.6 log_10_) of PV type 3 (Saukett strain) on plastic surfaces after a 1-min contact time. Tyler et al. [14] also found sodium hypochlorite to be reproducibly effective in completely inactivating PV on glass surfaces, being unable to recover infectious virus from the glass surface after a 5- or 10-min contact time with 2500 or 9200 ppm sodium hypochlorite. The repeatability associated with the efficacy of 5000 ppm sodium hypochlorite for inactivating PV-1 on stainless-steel surfaces in our study is shown in Table 3. The inactivation approach involving 5000 ppm sodium hypochlorite applied to contaminated surfaces for 5 min would appear to have the requisite efficacy and repeatability, and when applied at a 5-min contact time should have an adequate safety margin with respect to contact time for the GAPIV application.

Sodium hypochlorite, while representing an effective option for decontaminating PV-contaminated surfaces, has some surface compatibility issues, especially for stainless-steel [19]. For this reason, use of sodium hypochlorite for decontaminating stainless-steel surfaces is typically followed up with an alcohol wipe to remove the bleach residue. In the second portion of our study, we evaluated the time kinetics and repeatability of 95% ethanol as a stand-alone PV-decontamination approach (Figure 1b and Table 3). The time course/repeatability study indicated that the 3- and 5-min contact times led to reproducibly complete (≥4.5 to ≥4.7 log_10_) PV inactivation. As mentioned above, this result differs from the results reported by Tyler et al. [14], who reported that the PV inactivation by absolute ethanol was incomplete for contact times as high as 10 min. The differences in results are believed to be methodological. We have been unable to identify other studies in the literature for 95% or absolute ethanol inactivation of PV on surfaces. On the basis of our results, 95% ethanol applied as a soak onto PV-contaminated stainless-steel surfaces for 5 min would appear to represent an effective option for decontaminating surfaces in a GAPIV [2] setting.

The limitations of our study included the following: First, we did not evaluate each of the possible physical or chemical PV-inactivation approaches available. We did, however, select for final evaluation inactivation approaches which should be available within laboratories globally. Second, we did not challenge the candidate inactivation approaches with PV types 2 and 3 or any of the vaccine variants. However, the data of Orel et al. [18] suggest that differences in the efficacy of chemical inactivation approaches within PV strains are not to be expected. Third, we examined only stainless-steel carriers. Previous work [14,17,18] suggests that similar PV-inactivation efficacies are to be expected for other non-porous surfaces such as glass and plastic. The inactivation efficacies for PV on porous surfaces, however, are less clear. Fourth, we were not able to challenge the candidate inactivation approaches with PV titers above 6.9 TCID_50_/mL. This is a methodological constraint that we are not able to overcome for PV at this time.

## 5. Conclusions

While the EPA [3] and Health Canada [6] require at least a 3 log_10_ inactivation of challenge virus for the registration of virucidal claims for surface-disinfecting products, for decontaminating surfaces for PV containment purposes in PEFs, we expect that greater log_10_ reductions will be desired. In addition, safety margins involving greater contact times than necessary to achieve complete PV inactivation should be expected. Two PV-inactivation approaches that should be available in PEF laboratories globally have been characterized for efficacy, time kinetics, and repeatability for decontaminating PV type 1 Chat strain on stainless-steel surfaces at room temperature. The approaches include application for 5 min of 5000 ppm sodium hypochlorite and 95% *v*/*v* ethanol in distilled water. We now report that both PV-inactivation approaches fulfill the expectations expressed above, with 5000 ppm sodium hypochlorite reproducibly causing ≥5.38 log_10_ inactivation and 95% ethanol reproducibly causing ≥4.46 log_10_ inactivation of PV within a 5-min contact time.

## Figures and Tables

**Table 1:** Virucidal treatments evaluated in this study ^a^.

Group	Virucidal Treatment (Contact Time)
1 (*n* = 1)	UV-C (15 min; ~139 mJ/cm^2^), ^b^ direct soak with Cavicide (5 min), spray with 70% isopropyl alcohol
	direct soak with Cavicide (5 min)
	direct soak with freshly prepared 10,000 ppm sodium hypochlorite in sterile distilled water (5 min)
2 (*n* = 1)	direct soak with freshly prepared 80% *v*/*v* ethanol in sterile distilled water (5 min)
3 (*n* = 1)	direct soak with freshly prepared 95% *v*/*v* ethanol in sterile distilled water (5 min)
	direct soak with freshly prepared 5000 ppm sodium hypochlorite (5 min)
	direct soak with freshly prepared 1000 ppm sodium hypochlorite (5 min)
4 (*n* = 3)	spray with freshly prepared 95% *v*/*v* ethanol (0.25, 1, 3, 5 min)
	spray with freshly prepared 5000 ppm sodium hypochlorite (0.25, 1, 3, 5 min)
5 (*n* = 2)	direct soak with aged (30 days capped at ambient temperature) 95% *v/v* ethanol in sterile distilled water (1 min)
	direct soak with aged (30 days capped at ambient temperature) 95% *v/v* ethanol in sterile distilled water (5 min)

^a^ Direct soak means immersion of the carrier in the microbicidal solution; spray means spray from a distance of 6 to 8 inches until the carrier surface is completely wet; *v*, volume; ppm, parts per million. ^b^ A 60-inch 30 W germicidal UV-C lamp was used at a distance of ~61 cm from the carrier. The intensity at the carrier surface was calculated to be 0.154 mW/cm^2^ per second. A 15 min exposure was applied, yielding a calculated UV fluence of 139 mJ/cm^2^. No dosimetry was performed to confirm this calculated fluence.

**Table 2:** Initial screening evaluation of inactivation approaches for poliovirus type 1 Chat strain.

Inactivation Approach ^1^	Concentration	Contact Time (Minutes)	Plate Recovery Control Load ^3^ (log_10_ TCID_50_)	Treated Carrier Load (log_10_ TCID_50_)	Log_10_ Reduction
Cavicide soak	see Table 1	5	6.85	4.85	2.00
UV-C ^2^ + Cavicide soak + isopropyl alcohol spray	See Table 1	See Table 1	6.85	4.03	2.82
Sodium hypochlorite soak	1000 ppm	5	6.60	4.60	2.00
	5000 ppm	5	6.60	≤2.10	≥4.50 ^4^
	10,000 ppm	5	6.85	≤3.10	≥3.75 ^4^
Ethanol soak	80% *v*/*v*	5	6.85	4.85	2.00
	95% *v*/*v*	5	6.60	≤2.10	≥4.50 ^4^
	95% *v*/*v* aged 30 days	1	6.35	2.60, 2.35	3.75, 4.00
		5	6.35	≤2.10, ≤2.10	≥4.25, ≥4.25 ^4^

^1^ See Table 1 for the meanings of soak vs. spray. ^2^ Abbreviations used: ppm, parts per million; TCID_50_, Tissue Culture Infectious Dose 50%; UV-C, ultraviolet C; *v*, volume. ^3^ The plate recovery control load was calculated from the recovery of the Plate Recovery Control (see Section 2.3). ^4^ Inactivation was complete (no residual infectious virus was detected to the limit of detection of the assay) and, therefore, the log_10_ reductions depicted are to be interpreted as “≥” the values shown.

**Table 3:** Time kinetics and repeatability of inactivation approaches for poliovirus type 1 Chat strain.

Inactivation Approach ^1^	Concentration	Contact Time (Minutes)	Plate Recovery Load (log_10_ TCID_50_) ^3^	Treated Carrier Load (log_10_ TCID_50_)	Log_10_ Reduction ^4^
Sodium Hypochlorite spray	5000 ppm ^2^	0.25	6.48 ± 0.14	≤2.16, ≤2.16, ≤2.16	≥4.31, ≥4.31, ≥4.31 ^5^
		1	6.48 ± 0.14	≤2.16, ≤2.16, ≤2.16	≥4.31, ≥4.31, ≥4.31 ^5^
		3	6.79 ± 0.29	≤2.12, ≤2.12, ≤2.12	≥4.67, ≥4.67, ≥4.67 ^5^
		5	6.54 ± 0.14	≤1.16, ≤1.16, ≤1.16	≥5.38, ≥5.38, ≥5.38 ^5^
Ethanol spray	95% *v*/*v*	0.25	6.48 ± 0.14	5.87, 6.37, 6.12	0.60, 0.10, 0.35
		1	6.48 ± 0.14	3.62, 3.37, 3.37	2.85, 3.10, 3.10 ^6^
		3	6.79 ± 0.29	≤2.12, ≤2.12, ≤2.12	≥4.67, ≥4.67, ≥4.67 ^5,7^
		5	6.54 ± 0.14	≤2.08, ≤2.08, ≤2.08	≥4.46, ≥4.46, ≥4.46 ^5^

^1^ See Table 1 for the meaning of spray. ^2^ Abbreviations used: min, minutes; ppm, parts per million; TCID_50_, Tissue Culture Infectious Dose 50%; *v*, volume. ^3^ The plate recovery load was calculated from the mean ± standard deviation of the *n* = 3 Plate Recovery Controls; see Section 2.3 ^4^ Three independent replicates were performed for each condition to assess repeatability. ^5^ Inactivation was complete (no residual infectious virus was detected to the limit of detection of the assay), and, therefore, the log_10_ reductions depicted are to be interpreted as “≥” the values shown. ^6^ The difference between the mean 1 min and mean 0.25 min log_10_ reduction is statistically significant (*p* < 0.01 by a two-tailed T-test). ^7^ The difference between the mean 3-min and mean 1-min log_10_ reduction is statistically significant (*p* < 0.01 by a two-tailed T-test).

## Data Availability

Data are contained within the article and Supplementary Materials.

## Supplementary Materials

The following supporting information can be downloaded at https://www.mdpi.com/article/10.3390/microorganisms12101964/s1: Table S1: Interference and cytotoxicity testing.
