# Supplementary material for: Efficacy of Virucidal Treatments against Poliovirus Type 1 Deposited on Stainless-Steel Carriers
Source: Microorganisms. 2024 Sep 27;12(10):1964. doi: 10.3390/microorganisms12101964 (PMC11509370; doi:10.3390/microorganisms12101964)
Supplement: Supplementary file 1 [file microorganisms-12-01964-s001.zip › microorganisms-3205817-supplementary.pdf]

Supplementary Materials

Efficacy of virucidal treatments against poliovirus type 1 deposited on stainless-steel carriers

Cory Chiossone, Tanya Kapes, Robert Good, Raymond W. Nims, S. Steve Zhou

1. Evaluation of the cytotoxic effects of the test microbicides and the neutralizer used.

For the neutralizer effectiveness/viral interference and cytotoxicity controls, one replicate was performed for each test substance. This control was performed in the same manner as in the efficacy test, using dilution media in place of virus. Samples were neutralized 1:1 with the neutralizer and this post-neutralized sample (considered undiluted) was divided into two portions. Both portions were ten-fold serially diluted in dilution medium. For the cytotoxicity control, these dilutions were inoculated onto host cells. For the neutralization effectiveness/viral interference testing (NE/VI), the diluted samples (4.5 mL) were spiked with 100 µL of low titered virus (containing no more than approximately 5,000 $TCID_{50}$ units) and were held for at least the contact time. After holding, the dilutions were plated onto host cells.

Table S1. Interference and cytotoxicity testing (consisting of Microbac Tables 6-15)

Results for Poliovirus Disinfection R&Ds

Table 6
Neutralizer Effectiveness/Viral Interference (NE/VI) and Cytotoxicity (CT) Controls - 10,000 ppm Sodium Hypochlorite

Dilution*	NE/VI	CT
10^{0}	Cytotoxicity observed in all inoculated wells	Cytotoxicity observed in all inoculated wells
10^{-1}	Cytotoxicity observed in all inoculated wells	Cytotoxicity observed in all inoculated wells
10^{-2}	virus detected in all inoculated wells	no virus detected in all inoculated wells

* Dilution refers to the fold of the diliuton from the neutralized sample.

Table 7
Neutralizer Effectiveness/Viral Interference (NE/VI) and Cytotoxicity (CT) Controls - Cavicide

Dilution*	NE/VI	CT
10^{0}	Cytotoxicity observed in all inoculated wells	Cytotoxicity observed in all inoculated wells
10^{-1}	Cytotoxicity observed in all inoculated wells	Cytotoxicity observed in all inoculated wells
10^{-2}	virus detected in all inoculated wells	no virus detected in all inoculated wells

* Dilution refers to the fold of the diliuton from the neutralized sample.

Table 8
Neutralizer Effectiveness/Viral Interference (NE/VI) and Cytotoxicity (CT) Controls - UV 15 minutes, Cavicide 5 minutes, Isopropyl alcohol

Dilution*	NE/VI	CT
10^{0}	Cytotoxicity observed in all inoculated wells	Cytotoxicity observed in all inoculated wells
10^{-1}	Cytotoxicity observed in all inoculated wells	Cytotoxicity observed in all inoculated wells
10^{-2}	virus detected in all inoculated wells	no virus detected in all inoculated wells

* Dilution refers to the fold of the diliuton from the neutralized sample.

Table 9
Neutralizer Effectiveness/Viral Interference (NE/VI) and Cytotoxicity (CT) Controls - 80% Ethanol

Dilution*	NE/VI	CT
10^{0}	Cytotoxicity observed in all inoculated wells	Cytotoxicity observed in all inoculated wells
10^{-1}	virus detected in all inoculated wells	no virus detected in all inoculated wells
10^{-2}	virus detected in all inoculated wells	no virus detected in all inoculated wells

* Dilution refers to the fold of the diliuton from the neutralized sample.

Results for Poliovirus Disinfection R&Ds

Table 10
Neutralizer Effectiveness/Viral Interference (NE/VI) and Cytotoxicity (CT) Controls - 1,000 ppm Sodium Hypochlorite

Dilution*	NE/VI	CT
10^0	Cytotoxicity observed in all inoculated wells	Cytotoxicity observed in all inoculated wells
10^{-1}	virus detected in all inoculated wells	no virus detected in all inoculated wells
10^{-2}	virus detected in all inoculated wells	no virus detected in all inoculated wells

* Dilution refers to the fold of the diliuton from the neutralized sample.

Table 11
Neutralizer Effectiveness/Viral Interference (NE/VI) and Cytotoxicity (CT) Controls - 5,000 ppm Sodium Hypochlorite

Dilution*	NE/VI	CT
10^0	Cytotoxicity observed in all inoculated wells	Cytotoxicity observed in all inoculated wells
10^{-1}	virus detected in all inoculated wells	no virus detected in all inoculated wells
10^{-2}	virus detected in all inoculated wells	no virus detected in all inoculated wells

* Dilution refers to the fold of the diliuton from the neutralized sample.

Table 12
Neutralizer Effectiveness/Viral Interference (NE/VI) and Cytotoxicity (CT) Controls - 95% Ethanol

Dilution*	NE/VI	CT
10^0	Cytotoxicity observed in all inoculated wells	Cytotoxicity observed in all inoculated wells
10^{-1}	virus detected in all inoculated wells	no virus detected in all inoculated wells
10^{-2}	virus detected in all inoculated wells	no virus detected in all inoculated wells

* Dilution refers to the fold of the diliuton from the neutralized sample.

Results for Poliovirus Disinfection R&Ds

Table 13
Neutralizer Effectiveness/Viral Interference (NE/VI) and Cytotoxicity (CT) Controls - 5,000 ppm Sodium Hypochlorite (05/19/23)

Dilution*	NE/VI	CT
10^0	Cytotoxicity observed in all inoculated wells	Cytotoxicity observed in all inoculated wells
10^{-1}	virus detected in all inoculated wells	no virus detected in all inoculated wells
10^{-2}	virus detected in all inoculated wells	no virus detected in all inoculated wells

* Dilution refers to the fold of the diliuton from the neutralized sample.

Table 14
Neutralizer Effectiveness/Viral Interference (NE/VI) and Cytotoxicity (CT) Controls - 95% Ethanol (05/19/23)

Dilution*	NE/VI	CT
10^0	Cytotoxicity observed in all inoculated wells	Cytotoxicity observed in all inoculated wells
10^{-1}	virus detected in all inoculated wells	no virus detected in all inoculated wells
10^{-2}	virus detected in all inoculated wells	no virus detected in all inoculated wells

* Dilution refers to the fold of the diliuton from the neutralized sample.

Table 15
Neutralizer Effectiveness/Viral Interference (NE/VI) and Cytotoxicity (CT) Controls - 95% Ethanol - aged 30 days

Dilution*	NE/VI	CT
10^0	Cytotoxicity observed in all inoculated wells	Cytotoxicity observed in all inoculated wells
10^{-1}	virus detected in all inoculated wells	no virus detected in all inoculated wells
10^{-2}	virus detected in all inoculated wells	no virus detected in all inoculated wells

* Dilution refers to the fold of the diliuton from the neutralized sample.
